# Comprehensive analysis of CXCR family members in lung adenocarcinoma with prognostic values

**DOI:** 10.1186/s12890-022-02051-6

**Published:** 2022-06-29

**Authors:** Lian-Tao Hu, Wen-Jun Deng, Zhen-Sheng Chu, Luo Sun, Chun-Bin Zhang, Shi-Zhen Lu, Jin-Ru Weng, Qiao-Sheng Ren, Xin-Yu Dong, Wei-Dong Li, Xue-Bin Li, Yun-Ting Du, Yue Li, Wei-Qun Wang

**Affiliations:** 1 Basic Medical College, Jiamusi University, Jiamusi, 154002 Heilongjiang China; 2 Key Laboratory of Microecology-Immune Regulatory Network and Related Diseases, Jiamusi, 154002 Heilongjiang China; 3 First Affiliated Hospital, Jiamusi University, Jiamusi, 154002 Heilongjiang China; 4 Stomatological Hospital, Jiamusi University, Jiamusi, 154002 Heilongjiang China; 5 Department of Medical Technology, Collaborative Innovation Center for Translation Medical Testing and Application Technology Zhangzhou, Zhang Zhou Health Vocational College, Zhangzhou, 363000 Fujian Province China; 6 Department of Laboratory, Cancer Hospital of China Medical University, Shenyang,, Liaoning Province China

**Keywords:** CXC chemokine receptors (CXCRs), Lung adenocarcinoma (LUAD), Prognostic values

## Abstract

**Background:**

The expression profiles and molecular mechanisms of CXC chemokine receptors (CXCRs) in Lung adenocarcinoma (LUAD) have been extensively explored. However, the comprehensive prognostic values of CXCR members in LUAD have not yet been clearly identified.

**Methods:**

Multiple available datasets, including Oncomine datasets, the cancer genome atlas (TCGA), HPA platform, GeneMANIA platform, DAVID platform and the tumor immune estimation resource (TIMER) were used to detect the expression of CXCRs in LUAD, as well as elucidate the significance and value of novel CXCRs-associated genes and signaling pathways in LUAD.

**Results:**

The mRNA and/or protein expression of CXCR1, CXCR2, CXCR3, CXCR4, CXCR5 and CXCR6 displayed predominantly decreased in LUAD tissues as compared to normal tissues. On the contrary, compared with the normal tissues, the expression of CXCR7 was significantly increased in LUAD tissues. Subsequently, we constructed a network including CXCR family members and their 20 related genes, and the related GO functions assay showed that CXCRs connected with these genes participated in the process of LUAD through several signal pathways including Chemokine signaling pathway, Cytokine-cytokine receptor interaction and Neuroactive ligand-receptor interaction. TCGA and Timer platform revealed that the mRNA expression of CXCR family members was significantly related to individual cancer stages, cancer subtypes, patient’s gender and the immune infiltration level. Finally, survival analysis showed that low mRNA expression levels of CXCR2 (HR = 0.661, and Log-rank *P* = 1.90e−02), CXCR3 (HR = 0.674, and Log-rank *P* = 1.00e−02), CXCR4 (HR = 0.65, and Log-rank *P* = 5.01e−03), CXCR5 (HR = 0.608, and Log-rank *P* = 4.80e−03) and CXCR6 (HR = 0.622, and Log-rank *P* = 1.85e−03) were significantly associated with shorter overall survival (OS), whereas high CXCR7 mRNA expression (HR = 1.604, and Log-rank *P* = 4.27e−03) was extremely related with shorter OS in patients.

**Conclusion:**

Our findings from public databases provided a unique insight into expression characteristics and prognostic values of CXCR members in LUAD, which would be benefit for the understanding of pathogenesis, diagnosis, prognosis prediction and targeted treatment in LUAD.

## Background

Lung cancer is the most common type of human malignancy and the leading cause of cancer death [1]. Non-small cell lung cancer (NSCLC) is a common type of lung cancer, accounting for approximately 90% of all malignancies of the lung [2]. The two most common subtypes of non-small cell lung cancer are adenocarcinoma (LUAD) and squamous cell carcinoma (LUSC), which account for 50% and 40% of the total, respectively [3, 4]. Lung cancer consists of subpopulations of cells or clones that have distinct molecular characteristics of tumors and therefore genetically characterize the malignant behavior of most malignant tumors. Clonal mutations in patients with LUAD may increase the probability of postoperative recurrence, implying a higher propensity for early development of metastasis in lung adenocarcinoma and increasing cancer heterogeneity. [5] Similarly there are studies on the mechanisms of drug resistance in cancer showing that resistance to some drugs in lung cancer patients is objectively present before treatment. [6, 7]

Chemokines are a class of cytokines associated with cellular secretion and structure; their initial discovery and most important role is to make up the extracellular matrix of tumors and have the ability to directly influence cancer cell proliferation and metastasis. [8–10] Among them, CXC chemokines are a 7-transmembrane G protein-coupled receptor protein localized on the cell membrane with two cysteine residues near the N terminus. [11, 12] CXCR binds to its cognate ligand, changes the conformation of the receptor, and then activates the coupled G protein to start the corresponding signaling pathway to act in the cell. Most CXCRs are typical receptors coupled to G proteins, but CXCR7 is an atypical receptor coupled to a β receptor, also known as atypical chemokine receptor 3 (ACKR3). [13] In addition, some CXCRs do not bind only one ligand; for example, CXCR2 can bind to multiple ligands to exert different effects. [14] Following the action of the corresponding ligands that bind CXCRs, the receptors are usually degraded or restored to the plasma membrane by clathrin-mediated endocytosis. [15] CXCRs facilitate communication links between the intracellular and extracellular microenvironments, which in turn affect cellular behaviors such as cellular transport, proliferation and invasion. [16, 17] In addition, tumor angiogenesis and tumor immunity have emerged as one of the research areas related to CXCR in recent years.

Although a series of studies have elucidated the significant prognostic roles of some CXCR members in LUAD, the whole picture of the prognostic values of CXCR members remain inequitably characterized in LUAD. In this study, the clinical significance of CXCR family members in LUAD was analyzed in terms of mRNA expression level, protein expression level, immunity, interaction network, pathway analysis, clinical and prognostic aspects by using multiple databases and platforms based on the study of Xiaojuan Li et al. [18] in order to provide a general overview and expansion of the value of CXCRs in LUAD.

## Methods

### Differential study of CXCRs at the transcriptional level

Oncomine (https://www.oncomine.org/resource/login.html) is an integrated online gene microarray database and data mining platform that provides peer review, powerful analysis methods, and a robust set of analytical capabilities to calculate gene expression levels. [19] During the analysis of differential mRNA expression in LUAD tissues and their corresponding normal lung gland tissues, we selected data for CXCRs by the following criteria: *P*-value < 0.05, fold change = 1.5, gene rank = 10%, the Benjamini and Hochberg method was used to correct the *P* value.

The LUAD data in the Cancer Genome Atlas (TCGA) database (https://portal.gdc.cancer.gov/ repository) were analyzed using the UALCAN (http://ualcan.path.uab.edu/) platform. The database is a more comprehensive information platform containing gene expression databases and corresponding clinical information data. [20] UALCAN is an open access database developed on multiple platforms such as TCGA. [21] In this study, the differences in CXCRs mRNA expression levels expressed in normal samples and lung adenocarcinoma tissues were analyzed by comparing two data sets, UALCAN platform and oncomine.

### Differentially study CXCRs at the protein level

HPA (https://www.proteinatlas.org/) is a platform containing Protein expression level data for a wide range of common cancers. [22] Immunohistochemistry (IHC) staining data of all CXCRs were obtained from the HPA database and analyzed. In the HPA database, protein expression scoring was evaluated by taking among staining, intensity and quantity, including not detected (negative, none), low (weak, < 25%), medium (moderate, 25–75%), and high (strong, > 75%).

### Construction of protein interaction networks

GeneMANIA (http: //www.genemania.org/) is an interactive, visual online protein interaction prediction tool. [23] Given a list of gene queries, GeneMANIA uses available genomics and proteomics data to search for functionally similar genes to predict interacting genes for the target gene. In order to obtain the genes interacting with CXCRs and to construct a protein interaction network, seven CXCRs were studied as a whole in this study at GeneMANIA.

### GO enrichment analysis and kyoto encyclopedia of genes and genomes (KEGG) pathway enrichment analysis

DAVID (https://david.ncifcrf.gov/) is a functional enrichment analysis web tool with continuously updated and effectively reduce data redundancy. [24] In this study the biological processes, cellular components, molecular functions and Kyoto genes and genomic encyclopedia pathways involved in CXCR and the 20 genes associated with it were obtained by enrichment using DAVID, and then the results were visualized by creating enrichment bubble maps using R software.

### Correlation analysis of CXCRs and immune infiltration

TIMER (https://cistrome.shinyapps.io/timer/) is an online tool for systematical analyses of immune infiltration of various cancers. [25] Then the correlation between the six immune cells and CXCR was analyzed by this tool. Their required correlations and *P*-values were automatically calculated and displayed in the graphs. And the Benjamini and Hochberg method was used to correct the P value.

### Clinicopathological analysis associated with CXCRs

In addition, downloaded TCGA data were used to analyze the association between mRNA expression of CXCRs in LUAD tissues and their clinicopathological parameters (e.g., TNM stage, cell subtype, and gender of the patient). The results could be got by selecting R (4.02) integrated into the TCGA database.

## Survival analysis

The TCGA-LUAD gene expression data and clinical data were read separately using R (v4.0.2) software, and the patients were divided into high and low expression groups using the median expression of the samples as the cut-off point. The "survival" package was applied to analyze the survival rate of the high and low gene expression groups. *P* < 0.05 was considered statically significant.

## Results

### MRNA expression of CXCR family members in LUAD patients

**Fig. 1:**
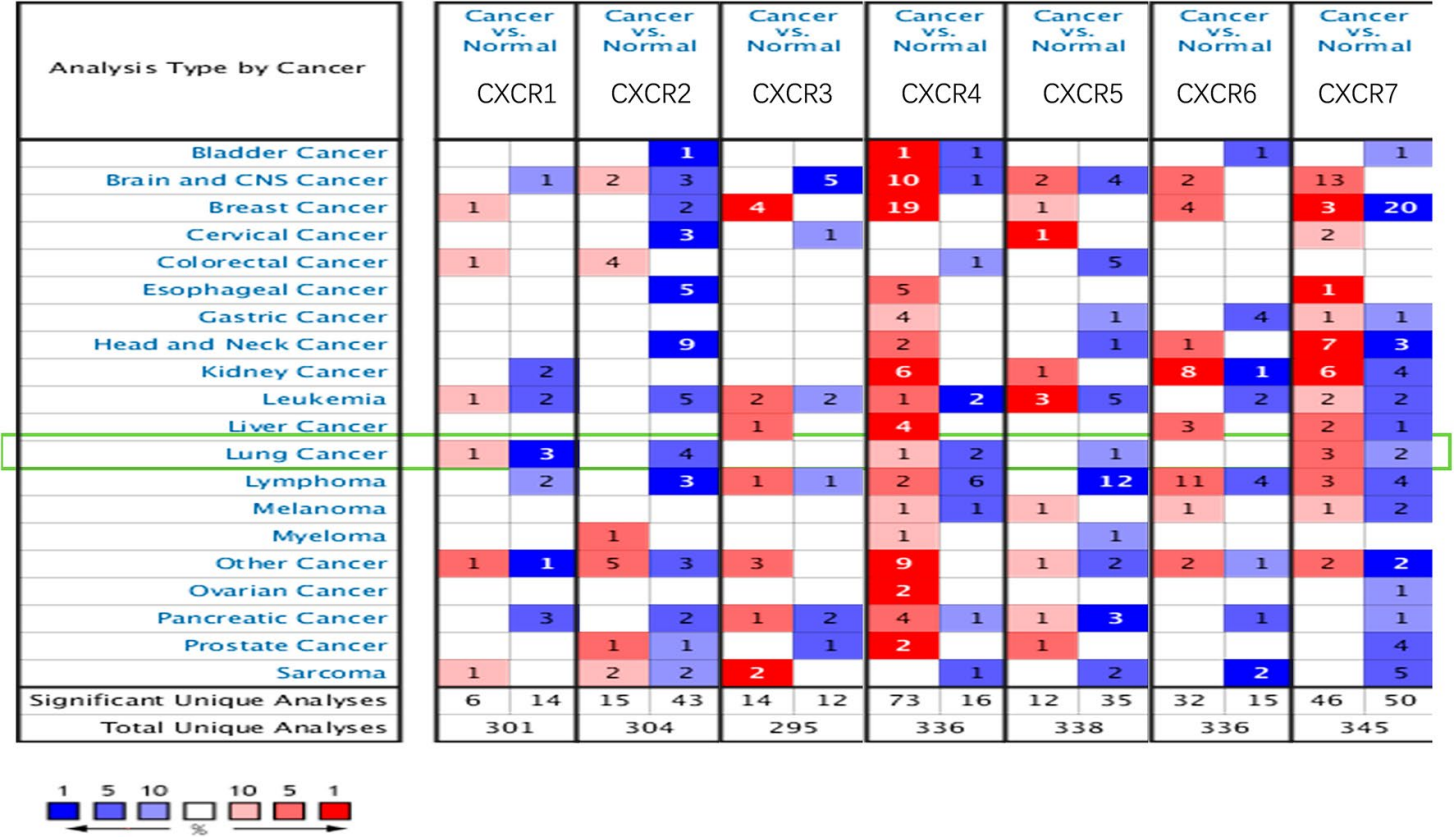
Transcriptional expressions of different CXCR family members in different types of cancers. The data were compared by the t-test and cut-off *P*-value and fold change were as following: *P*-value < 0.05, fold change = 1.5, gene rank = 10%. (Red indicates over-expression, blue indicates down-expression)

This study first used the Oncomine database to compare CXCRs mRNA expression levels from multiple cancers, and then to identify differences in CXCRs expression in LUAD patients. By comparing the expression of CXCRs in multiple cancer species, Oncomine data visualization results showed differences in their mRNA expression levels in LUAD patients. (Fig. 1) The differential expression of CXCRs in different subtypes of lung cancer was then obtained by pooling data from various sources in Oncomine as follows. The mRNA expression levels of CXCR1 (*P* = 4.71E−7, *P* = 1.19E−6), CXCR2 (*P* = 4.20E−16, *P* = 1.76E−11), CXCR4 (*P* = 1.63E−15) and CXCR7 (*P* = 1.31E−12, *P* = 1.70E−8) were significantly lower in LUAD than in adjacent tissues. (Table 1).

**Table1:** CXCR1, CXCR2, CXCR4, CXCR5 and CXCR7 displayed significant regulated expression in lung cancer compared to normal from Oncomine

	Types of lung cancer versus normal	Fold change	*P*-value	t-test	FDR	Ref
CXCR1	Lung Carcinoid Tumor versus normal	3.098	0.006	2.741	0.006375	Bhattacharjee lung
	Squamous Cell Lung Carcinoma versus normal	− 6.385	4.58E−08	− 6.724	9.73E−08	Bhattacharjee lung
	Lung Adenocarcinoma versus normal	− 3.22	4.71E−07	− 6.221	8.01E−07	Bhattacharjee lung
	Lung Adenocarcinoma versus normal	− 2.073	1.19E−06	− 5.572	1.69E−06	Stearman lung
CXCR2	Large Cell Lung Carcinoma versus normal	− 4.492	1.17E−14	− 10.806	6.63E−14	Hou lung
	Lung Adenocarcinoma versus normal	− 3.109	4.20E−16	− 9.451	7.14E−15	Hou lung
	Squamous Cell Lung Carcinoma versus normal	− 3.426	1.19E−13	− 9.102	5.06E−13	Hou lung
	Lung Adenocarcinoma versus normal	− 1.536	1.76E−11	− 8.097	4.99E−11	Selamat lung
CXCR4	Squamous Cell Lung Carcinoma versus normal	2.676	6.13E−07	5.402	9.47E−07	Talbot lung
	Lung Adenocarcinoma versus normal	− 2.907	1.63E−15	− 9.17	1.39E−14	Selamat lung
	Lung Carcinoid Tumor versus normal	− 13.625	6.23E−08	− 7.866	1.18E−07	Bhattacharjee lung
CXCR5	Squamous Cell Lung Carcinoma versus normal	− 2.231	0.014	− 2.291	0.014	bhattacharjee lung
CXCR7	Squamous Cell Lung Carcinoma versus normal	6.403	6.46E−05	5.292	8.45E−05	Bhattacharjee lung
	Squamous Cell Lung Carcinoma versus normal	5.437	0.002	4.087	0.002429	Wachi lung
	Squamous Cell Lung Carcinoma versus normal	3.181	0.002	3.456	0.002267	Garber lung
	Lung Adenocarcinoma versus normal	− 2.587	1.31E−12	− 8.2	4.45E−12	Selamat lung
	Lung Adenocarcinoma versus normal	− 2.1	1.70E−08	− 7.304	4.13E−08	Okayama lung

**Fig. 2:**
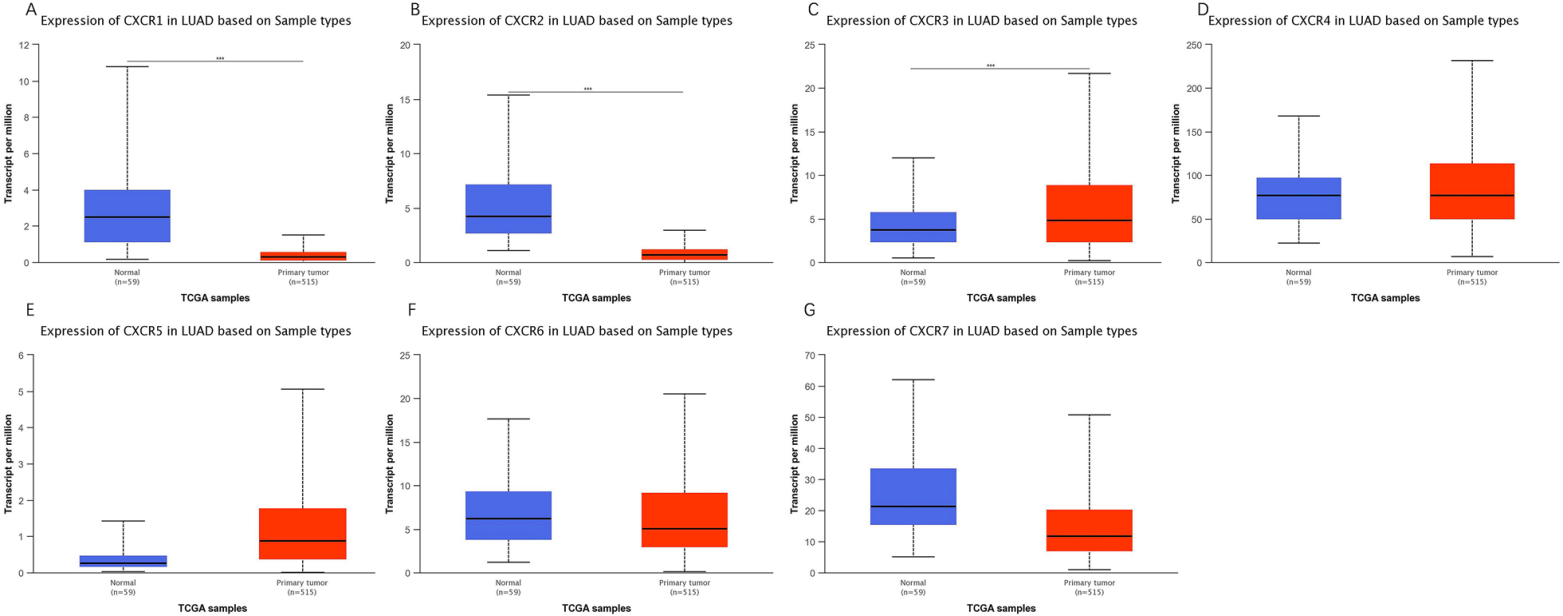
MRNA expressions of CXCR family members in patients with LUAD and normal lung tissues. The mRNA expressions of different CXCR family members were significantly regulated in patients with LUAD from the TCGA database (*P < 0.05, **P < 0.01, ***P < 0.001.)

The results derived from Oncomine were then validated by the UALCAN platform. The results showed that mRNA expression levels of CXCR1 and CXCR2 were significantly lower and CXCR3 was significantly upregulated in LUAD compared to adjacent tissues. (Fig. 2).

### Protein expression of CXCR family members in LUAD patients

**Fig. 3:**
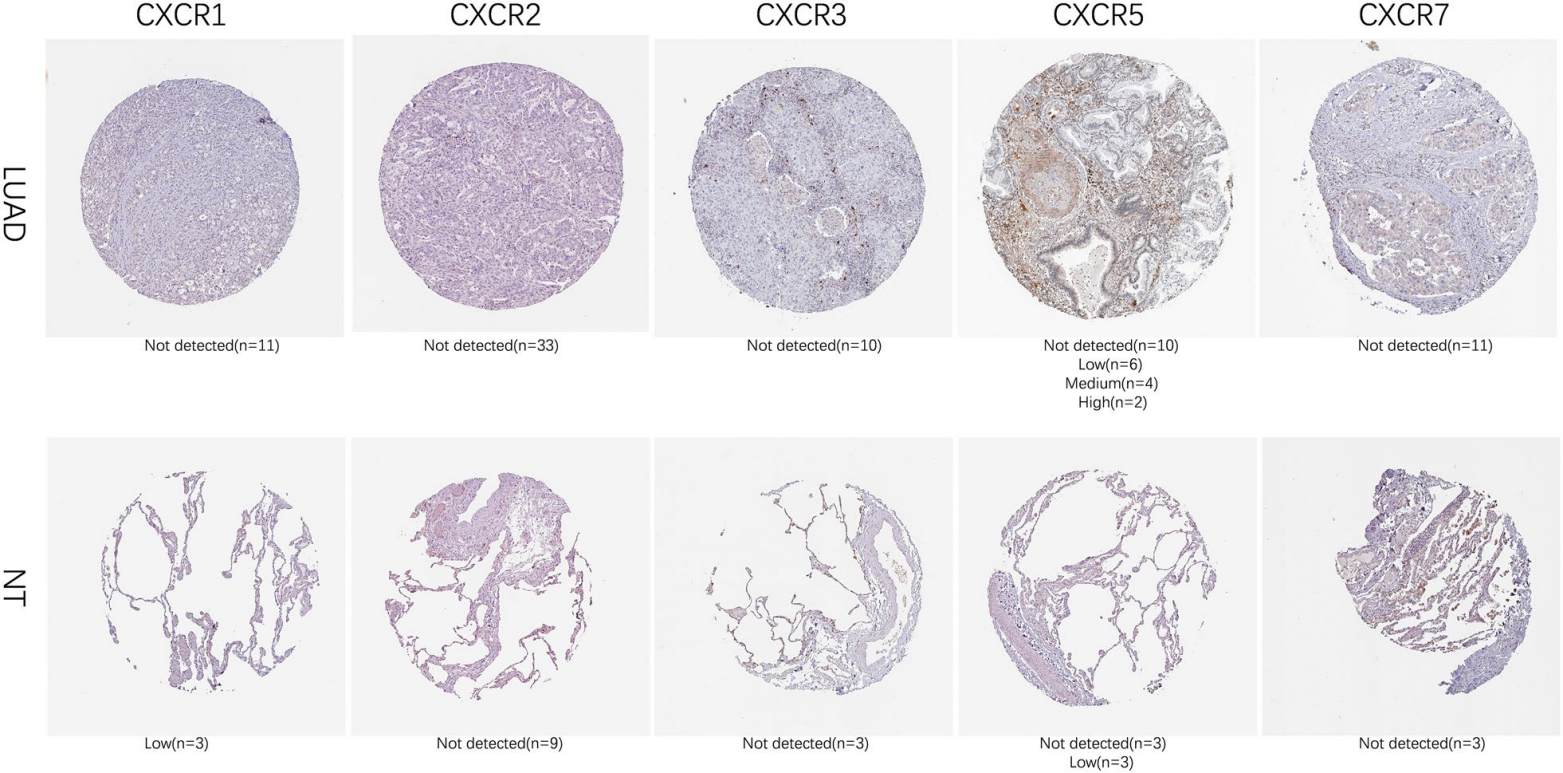
Protein expressions of CXCR family members in patients with NSCLC and normal lung tissues. Compared to normal samples protein expressions of CXCR1, CXCR3 and CXCR5 were significantly down-regulated and the protein expressions of CXCR7 was up-regulated

We then found that the protein expression of CXCR2, CXCR3 and CXCR7 was unchanged in LUAD compared to normal samples. The LUAD tissues displayed low (3/3) CXCR1 staining and the CXCR1 staining in lung adenocarcinoma cells was usually not detected (11/11). While the CXCR5 staining in lung alveolar cells was usually not detected (3/6) or low (3/6), the LUAD tissues displayed not detected (10/22), low (6/22), medium (4/22) or high (2/22) CXCR1 staining. (Fig. 3).

### Function enrichment of CXCR family members in LUAD

**Fig. 4:**
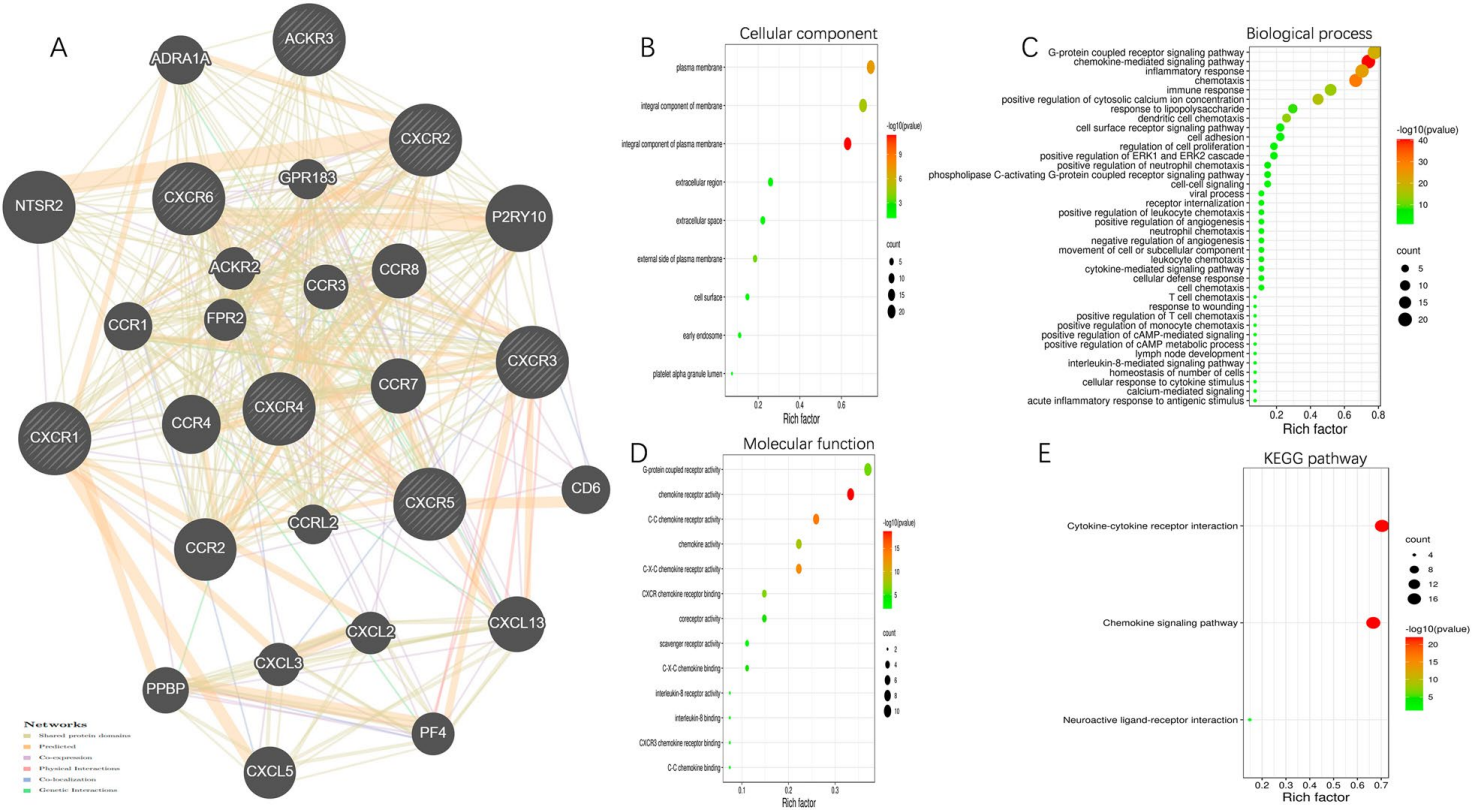
Function enrichment of CXCRs family members. **A** Network of CXCRs and their 20 related genes was analyzed. **B** Cellular component; **C** Biological processes; **D** Molecular functions; **E** KEGG pathway analysis

In this study, core proteins interacting with CXCR family members were identified through the GeneMANIA platform including NTSR2, P2RY10, CCR2, CCR4, CXCL13, CCR7, CCR8, CXCL5, ADRA1A, CD6, CCR1, PPBP, CCR3, CXCL3, PF4, ACKR2, CXCL2, FPR2, GPR183 and CCRL2. CXCL2, FPR2, GPR183, and CCRL2. and an interaction network was constructed using CXCR family members and their associated core genes to determine their relationships. For example, NTSR2 and CXCR7 share the same protein structural domain. CXCR1 and NTSR2 have a co-expression relationship. (Fig. 4A).

This study then performed GO function and pathway analysis of the above gene set via DAVID to predict the subsequent complex biological functions and signaling pathways. The biological processes such as G-protein coupled receptor signaling pathway, chemokine-mediated signaling pathway, inflammatory response, chemotaxis, immune response and positive regulation of cytosolic calcium ion concentration were remarkably regulated by the CXCRs in LUAD. (Fig. 4C) The cellular components including plasma membrane, integral component of membrane andintegral component of plasma membrane. (Fig. 4B) Besides, CXCRs also prominently affected the molecular functions, such as G-protein coupled receptor activity, chemokine receptor activity, C–C chemokine receptor activity, chemokine activity and C–X–C chemokine receptor activity were significantly associated with the CXCRs alterations. (Fig. 4D).

In KEGG analysis, we found that chemokine signaling pathways, cytokine–cytokine receptor interactions and neuroactive ligand—receptor interactions may have relevance to CXCR function in LUAD. (Fig. 4E).

### Correlation of mRNA expression levels of CXCRs with immune infiltration levels in patients with LUAD

By examining the level of immune infiltration in the TIMER database, this study found that the mRNA expression levels of all CXCR family members correlated significantly with tumor purity and neutrophils in LUAD. The mRNA expression of all CXCR family members except CXCR7 correlated with B cells, CD8 + T cells, macrophages and dendritic cells at significant levels. The mRNA expression levels of all CXCR family members except CXCR1 correlated statistically significant levels with CD4 + T cells. (Table 2) In summary, CXCR7 was not significantly associated with the level of immune cell infiltration, whereas the other CXCRs were significantly and positively associated with the level of immune cells.

**Table2:** The mRNA expression of CXCR family members was significantly related to the immune infiltration level in LUAD

	Variable	Partial.cor	*P*	FDR
CXCR1	Neutrophil	0.312869173	1.98E−12	3.59E−12
	Macrophage	0.249274699	2.64E−08	4.46E−08
	Purity	− 0.17824121	0.0000679	0.000100821
	CD8 + T cell	0.174818215	0.000105366	0.000147512
	Dendritic cell	0.130815254	0.003793499	0.004533694
	CD4 + T cell	0.015004873	0.741949968	0.757407259
	B Cell	0.001784853	0.968758625	0.968758625
CXCR2	Neutrophil	0.401796767	3.65E−20	9.40607E−20
	Macrophage	0.362872692	1.53E−16	3.13212E−16
	Dendritic cell	0.359210442	2.62E−16	5.12873E−16
	Purity	− 0.24072146	6.07E−08	9.60138E−08
	B Cell	0.175763794	0.000101446	0.000146202
	CD4 + T cell	0.167192614	0.00022009	0.000299567
	CD8 + T cell	0.166146667	0.000230738	0.000305572
CXCR3	CD4 + T cell	0.616363368	5.64E−52	6.91312E−51
	B Cell	0.552889075	4.27E−40	4.18497E−39
	Dendritic cell	0.509673127	1.27E−33	8.91821E−33
	Purity	− 0.43192631	7.19E−24	2.20133E−23
	Neutrophil	0.415634552	1.35E−21	3.66463E−21
	CD8 + T cell	0.390159307	3.72E−19	8.68728E−19
	Macrophage	0.151872771	0.000792204	0.000995333
CXCR4	Neutrophil	0.493085431	5.88E−31	2.87961E−30
	CD8 + T cell	0.453227131	4.82E−26	1.81743E−25
	Dendritic cell	0.44949262	1.21E−25	4.23868E−25
	Purity	− 0.43502367	3.16E−24	1.03374E−23
	B Cell	0.435695062	7.66E−24	2.20717E−23
	cd4 + t cell	0.378304775	6.48E−18	1.38037E−17
	Macrophage	0.342264407	8.94E−15	1.68419E−14
CXCR5	B Cell	0.686994134	7.67E−69	3.75798E−67
	CD4 + T cell	0.619713613	1.12E−52	1.82373E−51
	Purity	− 0.50532384	2.24E−33	1.36985E−32
	Dendritic cell	0.380243706	3.11E−18	6.91733E−18
	Neutrophil	0.312228712	2.21E−12	3.87109E−12
	CD8 + T cell	0.222945521	6.69E−07	1.02509E−06
	Macrophage	0.085788423	0.059039297	0.067277338
CXCR6	CD8 + T cell	0.665355089	1.45E−63	3.55571E−62
	Neutrophil	0.54568435	7.94E−39	6.48232E−38
	Dendritic cell	0.49958271	3.60E−32	1.95736E−31
	B Cell	0.483594828	9.78E−30	4.35855E−29
	Purity	− 0.47813846	1.39E−29	5.65979E−29
	CD4 + T cell	0.394199431	1.92E−19	4.70594E−19
	Macrophage	0.243594719	5.55E−08	9.05999E−08
CXCR7	Purity	− 0.15337305	0.00062504	0.000805973
	Neutrophil	0.14448762	0.001452866	0.001779761
	CD4 + T cell	0.117935319	0.009405926	0.01097358
	B Cell	0.081301054	0.073944372	0.082347142
	Macrophage	0.051428777	0.258296354	0.28125603
	CD8 + T cell	0.017849732	0.694372443	0.739657602
	Dendritic cell	0.015208289	0.737534643	0.768919096

### Correlation of mRNA expression levels of CXCRs with clinicopathological features in patients with LUAD

This study then analyzed the relationship between mRNA expression levels of CXCR family members and clinicopathological parameters of LUAD patients, including individual cancer stage, cancer subtype and patient gender, using TCGA samples.

**Fig. 5:**
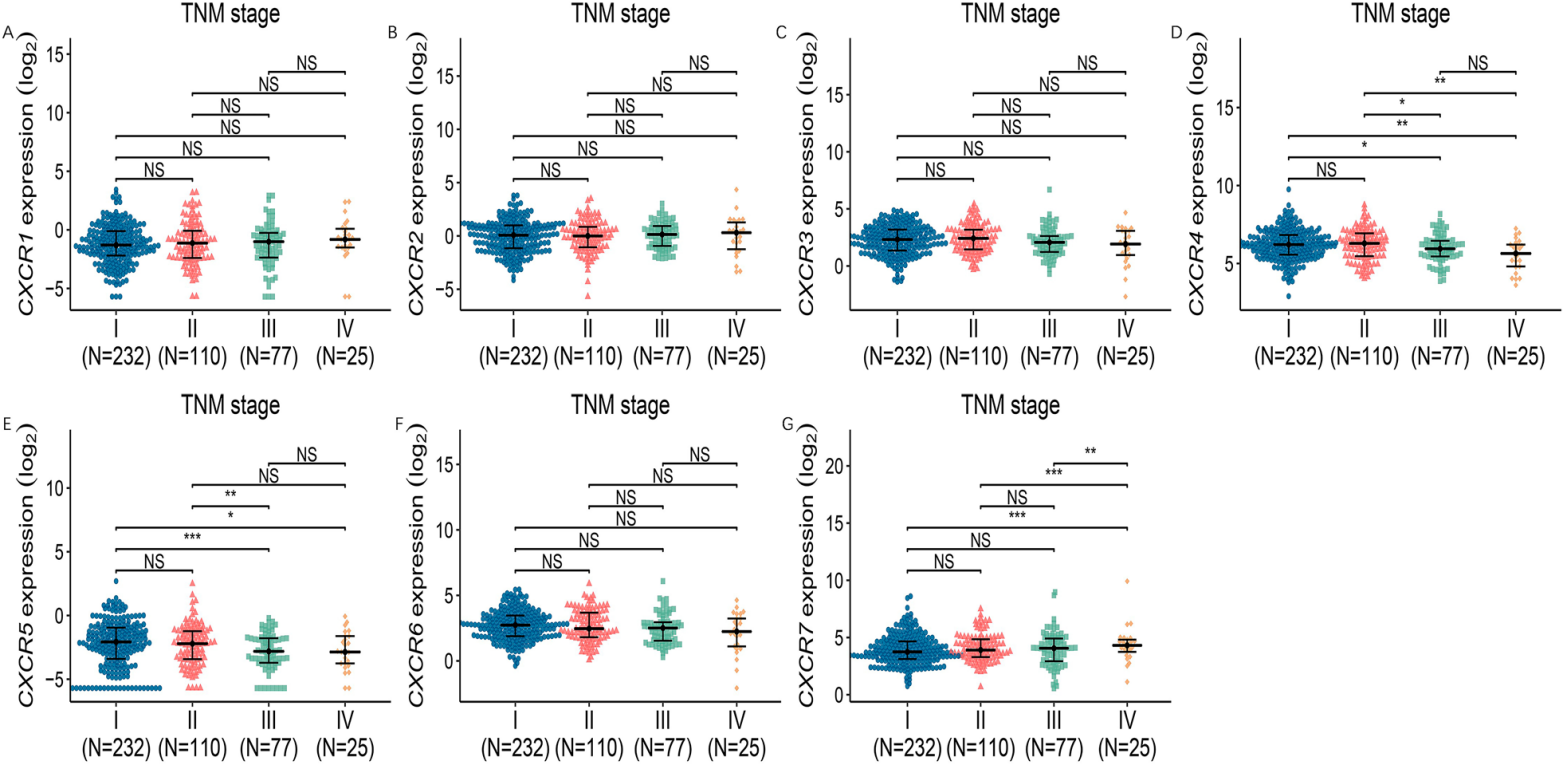
Association of mRNA expression of CXCRs family members with cancer stages of LUAD patients. The mRNA expression of CXCRs family members in normal people or in LUAD patients in different stages (*P < 0.05; **P < 0.01; ***P < 0.001)

**Fig. 6:**
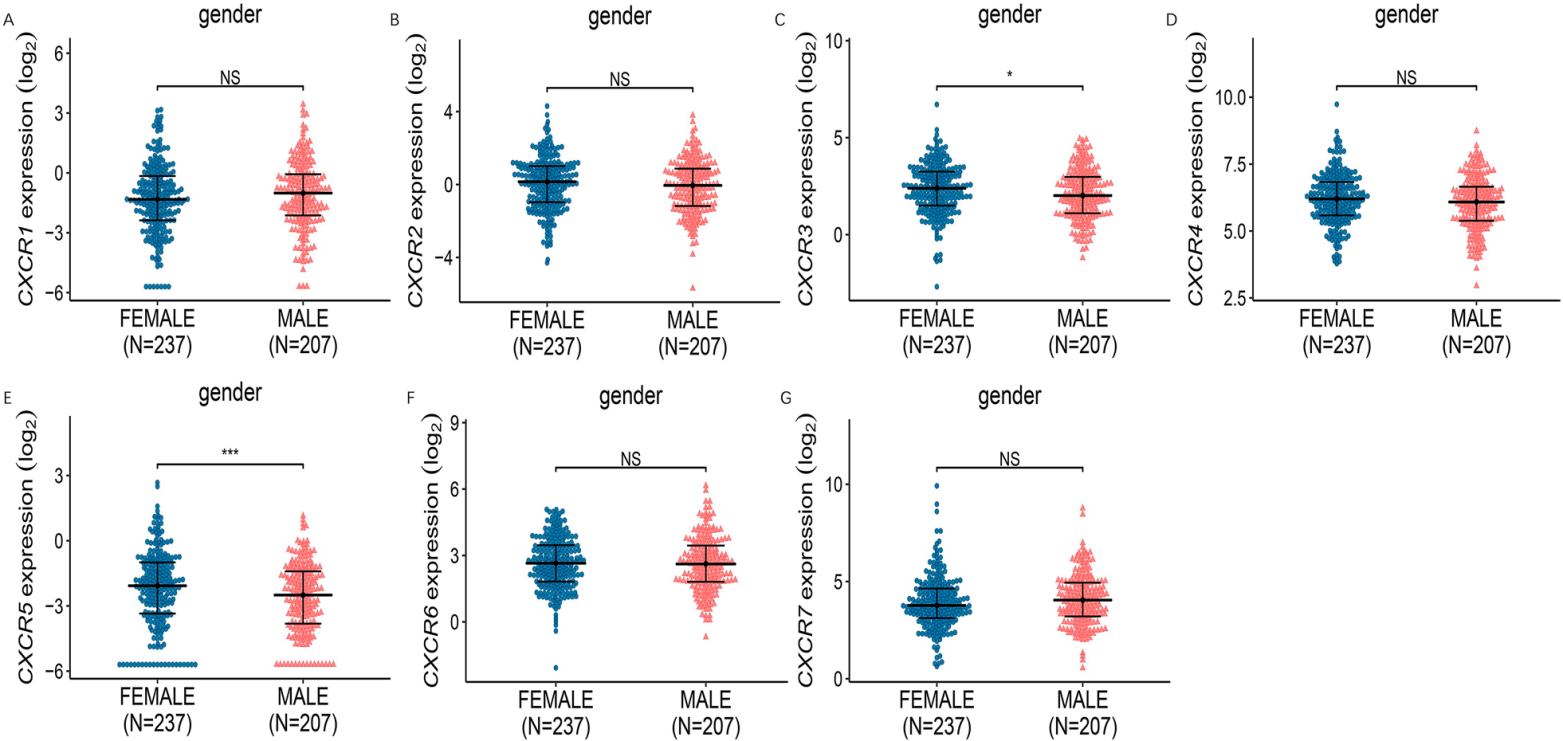
Association of mRNA expression of CXCRs family members with gender of LUAD patients. The mRNA expression of CXCR family members in normal people or in LUAD patients in male or female (*P < 0.05; **P < 0.01; ***P < 0.001)

The results of the analysis of TNM stage in LUAD patients showed that the mRNA expression levels of CXCR4, CXCR5 and CXCR6 gradually increased with the progression of TNM stage. Conversely, CXCR7 showed a gradual decrease in its expression level with the progression of TNM stage, while the difference between CXCR1, CXCR2 and CXCR3 in the progression of TNM stage was not significant. (Fig. 5) The results of analyzing the mRNA expression levels of CXCR family members in relation to the gender of LUAD patients showed that CXCR3 and CXCR5 had statistically significantly lower mRNA expression levels in males than in females in LUAD, while the differences between CXCR1, CXCR2, CXCR4, CXCR6 and CXCR7 were not significant. (Fig. 6).

**Fig. 7:**
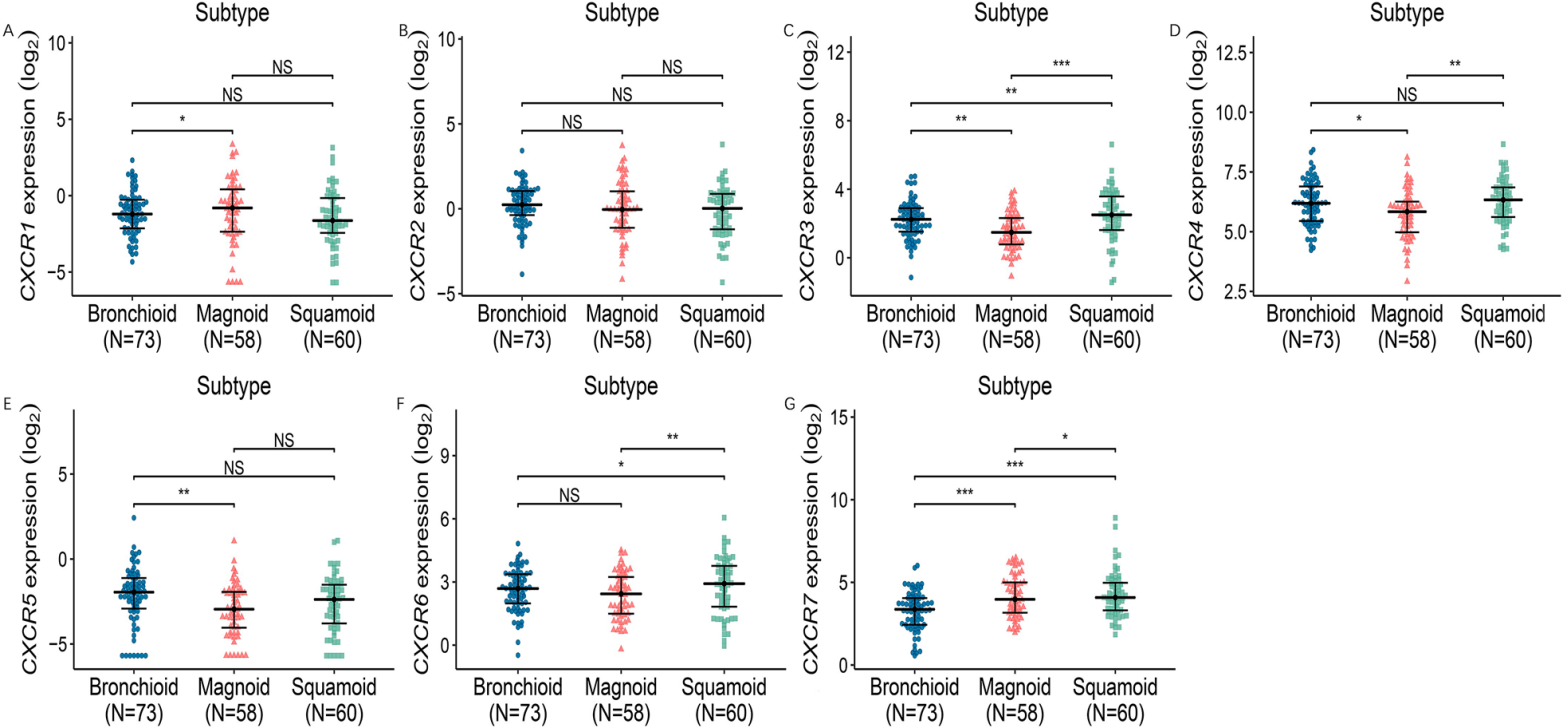
Association of mRNA expression of CXCR family members with cancer subtype of LUAD patients. The mRNA expression of CXCR family members in normal people or in LUAD patients in different subtype (*P < 0.05; **P < 0.01; ***P < 0.001)

The relationship between mRNA expressions of CXCR family members with cell subtypes of LUAD patients was analyzed by R. In LUAD, the expression of CXCR/3/4/5/6 in magnoid always lower than both bronchioid and squamoid. On the contrary, while CXCR7 in magnoid higher than both bronchioid and squamoid. (Fig. 7).

### Prognostic value of mRNA expression levels of family members in patients with LUAD

**Fig. 8:**
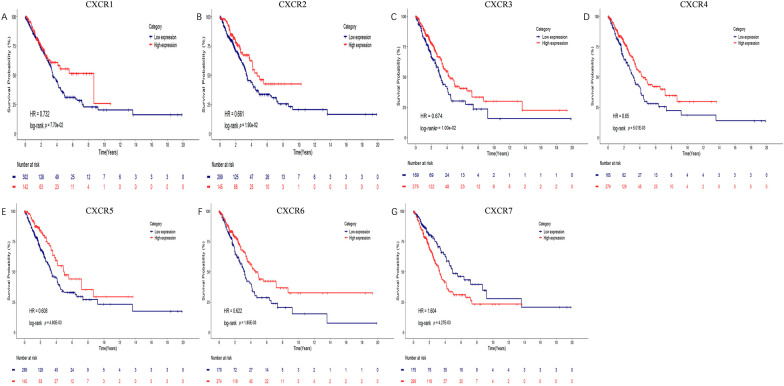
Prognostic value of mRNA expression of CXCR family members in LUAD patients about OS. OS comparing the high and low expression of CXCRs family members in LUAD patients

Kaplan–Meier analysis of the relationship between CXCRs and prognostic status of LUAD patients showed the following results. CXCR1/2/3///4/5/6 may play a role as beneficial factors in the prognosis of LUAD patients with HR values less than 1 and their *P* values of 7.70e−02, 1.90e−02, 1.00e−02, 5.01e−03, 4.80e−03, 1.85e−03, respectively. Conversely CXCR7 may play a role as a risk factor in the prognosis of LUAD patients with an HR of 1.604 and its *P* value of 4.27e−03. (Fig. 8) All of the above results suggest that the mRNA expression levels of CXCR family members are significantly associated with the prognosis of LUAD patients, with CXCR7 acting as a drug target for LUAD patients.

## Discussion

CXCRs is a superfamily of seven-transmembrane G protein-coupled receptors and play critical roles in immune surveillance, inflammation, tissue development and homeostasis. Increasing evidence suggested that various CXCRs were structural expression in various malignancies and be involved in the initiation, progression, and outcome of various human tumors.

In our study, we found that the low expression of CXCR1/2/3/4/5/6 in LUAD were associated with poor prognosis through TCGA dataset and multiple public databases, while low expression of CXCR7 was correlated with favorable survival in LUAD patients. We also studied the association between tumor immune infiltrating cells and CXCRs expression, and the results revealed that the expression levels of the seven CXCRs were significantly correlated with CD8 + T cell, CD4 + T cell, neutrophil, and dendritic cell, which were related to tumor progression, metastasis, or prognosis. [26]

The results of TCGA analysis showed that CXCR1 and CXCR2 were lowly expressed in LUAD, and patients with the cancer subtype of magnoid tended to express high level of CXCR1 mRNA. In addition to CXCL8, CXCR1 and CXCR2 were also activated by other CXC-chemokines, including CXCL1, − 2, − 3, − 5, − 6 and − 7. It is widely accepted that CXCR1 and CXCR2 are essential for the initiation and growth of human tumors, thus serve as the novel therapeutic targets for many solid tumors, including lung cancer, breast cancer, prostate cancer, ovarian cancer, liver cancer and melanoma. [27–30] However, our findings are inconsistent with these studies, the results from our enrichment and immune infiltration assays speculated that CXCR1 and CXCR2 may inhibit the progression of LUAD through their abilities to increase the level of immune activation. Given the current studies on CXCR1 or CXCR2 in LUAD remained sparse, CXCR1 and CXCR2 are worthy of further research and exploration.

We also found that the mRNA level of CXCR3 was higher, however, its protein level was down-regulated in LUAD. In addition, the mRNA expression of CXCR3 in female patients was significantly higher than that in male patients. Some evidence suggests that CXCR3 secreted by immune cells can inhibit the development of gastric cancer through paracrine pathway. [31] On the contrary, it was demonstrated that CXCR3 promotes the proliferation, migration and vascular invasion of cancer cells, such as breast cancer cells, gastric cancer cells. [32, 33] Thus, in different stages of tumor differentiation and development, we propose that CXCR3 plays different roles in the immune response of cancer cells.

In the current study, we also demonstrated that the expression levels of CXCR4/5 mRNA and protein were predominantly down-regulated in LUAD as compared to normal tissues, whereas there is no obvious difference in the expression of CXCR6 between the two groups. Furthermore, low expression of CXCR4/5 was significantly correlated with shorter survival, CXCR4/5 expression levels were also positively correlated with immune cell infiltration,and the expression levels of CXCR4 and CXCR5 were gradually decreased with the increase of TNM staging in patients with LUAD by TCGA dataset. Coupled with this, it was found that the overall survival ability of patients with lung cancer was significantly improved following the increase of CXCR4 expression in the tumor stroma. [34] A previous study also demonstrated that several leukocyte including recirculating B cells, small subsets of CD4 + and CD8 + T cells have the ability to express CXCR5, which in turn controls migration, entry and exit of these leukocytes to lymph nodes through interaction with its CXCL13 ligand. [35] In addition, invariant NKT (iNKT) cells is known to trigger potent antitumor responses in vivo due to their homeostasis and activation, in which CXCR6 and its ligand CXCL16 have been shown to play critical roles. [36] However, others suggested that CXCR4 and CXCR5 might play a significant role in the occurrence and development of breast cancer and prostate cancer. [37–39]

Our data also showed that the protein level of CXCR7 in LUAD was up-regulated compared with normal tissues, the high expression of CXCR7 was associated with poor OS, and the expression levels of CXCR7 increased gradually with the progress of TNM staging. Several studies have demonstrated that CXCR7 may be involved in tumor-associated signaling pathways, including PLC/MAPK, ERK1/2, STAT3 and AKT pathways, which have been revealed to play a prominent role in tumor cell adhesion, invasion and metastasis. [40–42]

To explore the potential mechanism of CXCR in LUAD, functional enrichment analyses were performed, according to the results of network, our results demonstrated that CXCRs and their related genes may be involved in the immune process, angiogenesis, and tumor initiation and progression via a variety of signaling pathways (e.g., Chemokine signaling pathway) and biological processes (e.g., immune response). According to the previous literature and our research results, we speculated that CXCRs, expressed by tumor cells and immune cells in the microenvironment, have the ability to regulate the growth, invasion and metastasis of LUAD in a variety of ways, in which the exact molecular mechanisms underlying these need to be further explored.

In summary, our results provided supportive evidence for undestanding the complexity of CXCR1-7 and their related biological functions, which may provide a valuable insight for the development of CXCRs-mediated targeted therapy. However, there were some limitations to our study. First, all the data was based on the online databases, further in vivo and in vitro studies are required to verify these findings. Second, the underlying mechanisms of CXCRs in LUAD is still unknown. In addition, the current study was only a retrospective study, future detailed prospective studies need to be further explored.

## Conclusions

Our findings from public databases provided a unique insight into expression characteristics and prognostic values of CXCR members in LUAD, which would be benefit for the understanding of pathogenesis, diagnosis, prognosis prediction and targeted treatment in LUAD.

## Data Availability

All data generated or analyzed in this study are contained in the multiple public databases mentioned in the article, including Oncomine (https://www.oncomine.org/resource/login.html), Human Protein Atlas (http://www.proteinatlas.org/), UALCAN (http://ualcan.path.uab.edu/), DAVID (https://david.ncifcrf.gov/), GeneMANIA (http://www.genemania.org/), TIMER (https://cistrome.shinyapps.io/timer/) and The Cancer Genome Atlas (TCGA) database (https://portal.gdc.cancer.gov/).

## Abbreviations

CXCR: CXC chemokine receptors
LUAD: Lung adenocarcinoma
TCGA: The cancer genome atlas
TIMER: The tumor immune estimation resource
OS: Overall survival
NSCLC: Non-small cell lung cancer
LUSC: Lung squamous cell carcinoma
GO: Gene ontology
KEGG: Kyoto encyclopedia of genes and genomes
TNM: Tumor node metastasis
CXCL: CXC chemokine ligands
NKT: Natural killer T cells
PLC: Phospholipase C
MAPK: Mitogen-activated protein kinase
ERK: Extracellular signal-regulated kinase
STAT3: Signal transducer and activator of transcription 3
AKT: Protein kinase B
